# Synthesis and Vasorelaxant and Platelet Antiaggregatory Activities of a New Series of 6-Halo-3-phenylcoumarins †

**DOI:** 10.3390/molecules15010270

**Published:** 2010-01-12

**Authors:** Elías Quezada, Giovanna Delogu, Carmen Picciau, Lourdes Santana, Gianni Podda, Fernanda Borges, Verónica García-Morales, Dolores Viña, Francisco Orallo

**Affiliations:** 1 Chemistry Department, Faculty of Sciences, University Porto, 4169-007 Porto, Portugal; E-Mails: elias.quezada@usc.es (E.Q.); mfernandamborges@gmail.com (F.B.); 2 Dipartimento Farmaco Chimico Tecnologico, Universita degli Studi di Cagliari, Via Ospedale 72, 09124 Cagliari, Italy; E-Mails: carmenpicciau@alice.it (C.P); gpodda@unica.it (G.P.); 3 Department of Organic Chemistry, Faculty of Pharmacy, University of Santiago de Compostela, 15782 Santiago de Compostela, Spain; E-Mail: lourdes.santana@usc.es (L.S.); 4 Department of Pharmacology, Faculty of Pharmacy, University of Santiago de Compostela, 15782 Santiago de Compostela, Spain; E-Mails: mdolores.vina@usc.es (D.V.); francisco.orallo@usc.es (F.O.); veronica.garcia9@rai.usc.es (V.G.M.)

**Keywords:** resveratrol, coumarin, vasorelaxant, platelet antiaggregatory activity

## Abstract

A series of 6-halo-3-hydroxyphenylcoumarins (resveratrol-coumarins hybrid derivatives) was synthesized in good yields by a Perkin reaction followed by hydrolysis. The new compounds were evaluated for their vasorelaxant activity in intact rat aorta rings pre-contracted with phenylephrine (PE), as well as for their inhibitory effects on platelet aggregation induced by thrombin in washed human platelets. These compounds concentration-dependently relaxed vascular smooth muscle and some of them showed a platelet antiaggregatory activity that was up to thirty times higher than that shown by *trans*-resveratrol and some other previously synthesized derivatives.

## 1. Introduction

**Figure 1:**
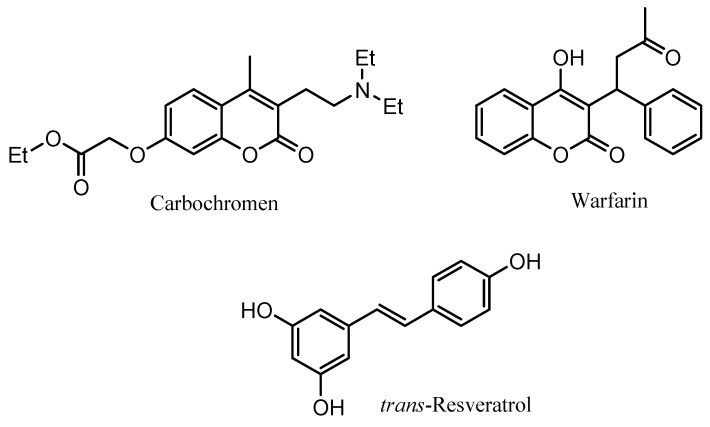
Chemical structures of *trans*-resveratrol, carbochromen, and warfarin.

Coumarins (or benzopyrones) are a large family of compounds of natural and synthetic origin that show numerous biological activities, including cardiovascular properties [1]. For instance, Carbochromen (3-diethylaminoethyl-7-ethoxycarbonylmethoxy-4-methylcoumarin (Figure 1) is a potent specific coronary vasodilator that has been used for many years in the treatment of angina pectoris [2,3]. Futhermore, warfarin [3-(2-acetyl-1-phenylethyl)-4-hydroxycoumarin (Figure 1) is a coumarin with potent anticoagulant activity and a good pharmacokinetic profile [4]. 

*trans*-Resveratrol (*t*-RESV; 3,4’,5-trihydroxy-*trans*-stilbene; Figure 1) is a natural phenolic component of *Vitis vinifera L.* (Vitaceae). It is abundant in the skin of the grapes and it is present in higher concentrations in red than in white wines. *trans*-Resveratrol has shown a number of biological activities, including protection against coronary heart disease, as a result of different effects: significant antioxidant activity, modulation of lipoprotein metabolism, and vasodilatatory and platelet antiaggregatory properties [5,6,7,8].

Because of their similar characteristics, it was of interest to design and synthesize hybrids that incorporate the nucleus of the coumarins and resveratrol molecules. In previous work, our research group had reported the vasorelaxant and platelet antiaggregatory activities of a series of coumarin-resveratrol hybrids (3-arylcoumarins), bearing hydroxy or methoxy groups on the coumarin and/or on the 3-phenyl ring. 6-Hydroxy-3-(3’,5’-dihydroxyphenyl)coumarin showed vasorelaxant and platelet antiaggregatory activity higher than that of *trans*-resveratrol [9].

Based on this, and with the aim of improving the vasorelaxant and platelet antiaggregatory activities of resveratrol-coumarin hybrids and establishing a relationship between the structure and activity for this type of compounds we have studied the effects of substitution on the 3-arylcoumarin moiety with groups showing different steric and electronics effects. In this paper we report the synthesis and evaluation of a new series of 3-arylcoumarins in which the hydroxy group in the 6 position has been changed for a halogen group, and different positions of hydroxyl group in 3-phenyl ring were explored.

## 2. Results and Discussion 

**Scheme 1:**
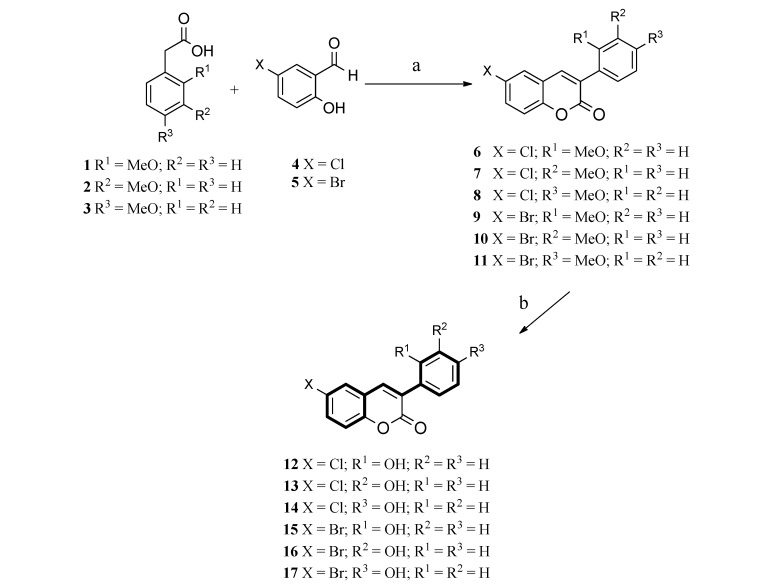
General synthetic route to obtain compounds **6-17.**

The 3-phenylcoumarins **6-11** [10] were prepared from the conveniently substituted phenylacetic acids **1-3**, the appropriate salicylaldehyde **4**, **5** and dicyclohexylcarbodiimide (DCC) by a Perkin reaction [11,12,13] in dimethylsulfoxide (DMSO). These reactions gave 46%, 40%, 27%, 29%, 31% and 33% yield, respectively. Hydrolysis of the methoxy groups [14] by treatment with HI in acetic acid/acetic anhydride gave the hydroxy derivatives **12**, **13**, **14**, **15**, **16** and **17** [15,16], in 28%, 70%, 60%, 73%, 94% and 57% yield, respectively (Scheme 1).

The vasorelaxant effects of compounds **12**-**17** were studied on pre-contracted rat aortic rings with endothelium [6]. The cumulative addition of *t*-RESV or the new compounds (1-100 μM) caused a concentration-dependent relaxation of the contractions induced by phenylephrine (PE, 1 μM) in intact rat aortic rings. The corresponding IC_50_ values are shown in Table 1.

All evaluated compounds resulted less efficient than *t*-RESV in relaxing the contractions induced by PE. Furthermore, substitution with a *p*-hydroxy group in the 3-phenyl ring (compounds **14** and **17**) leads to inactive compounds. Platelet aggregation studies were also performed. Compounds **12**, **13** and **17** inhibited platelet aggregation more effectively than *t*-RESV when thrombin (0.25 U/mL) was used as the stimulating agent (Table 2).

These results indicate that the variation of the position of the hydroxyl group on the 3-phenyl and the substitution with different halogen groups in the 6 position of the coumarin ring, in this type of molecules (resveratrol-coumarins hybrid), can give derivatives with a significantly higher pharmacological potency than *t*-RESV. This is the case for compound **13**, which has a platelet antiaggregatory activity that is more than 32 times higher than that of *t*-RESV.

## 3. Experimental

### 3.1. General

Melting points were determined in a Reichert Kofler thermopan or in capillary tubes in a Buchi 510 apparatus, and are uncorrected. Infrared (IR) spectra were recorded in a Perkin-Elmer 1640FT spectrometer (KBr disks, ν in cm-1).13C and 1H spectra of samples approximately 10% solutions in chloroform-d, were recorder at room temperature in 5 mm o.d. tubes. TMS was used as internal standard, chemical shifts are expressed in ppm (δ), J in Hz. NMR spectra were recorded with a Bruker AMX 500 (1H-, 500 MHz; 13C-, 125 MHz) instrument. ). Mass spectra were obtained using a Hewlett-Packard 5988A spectrometer (70 eV). Silica gel (35-60 mesh) was used for flash chromatography (FC). Analytical TLC was performed on plates precoated with silica gel (Merck 60 F254, 0.25 mm). Elemental analyses were performed with a Perkin-Elmer 240B microanalyser. Phenylacetic acids **1-3**, salicylaldehydes **4**, **5**, hydriodic acid (HI), acetic acid (AcOH), acetic anhydride (Ac_2_O), dicyclohexylcarbodiimide (DCC) and dimethylsulfoxide (DMSO) were commercially available (Aldrich).

### 3.2. Chemistry

The studied compounds are all known and were prepared by a traditional Perkin reaction carried out in refluxing dimethylsulfoxide (DMSO) between conveniently substituted phenylacetic acids **1-3** and the corresponding salicylaldehyde **4**, **5**, using dicyclohexylcarbodiimide (DCC) as dehydrating agent (Scheme 1) [11,12,13]. Hydrolysis of methoxy groups [14], by treatment with HI in acetic acid/acetic anhydride gave the desired hydroxyl derivatives **12-17** [15,16].

*6-Chloro-3-(2’-methoxy)phenylcoumarin* (**6**). Purified by chromatography using 9:1 hexane/ethyl acetate as eluent; Mp: 177–179 °C; IR (KBr): 2920, 1705, 1610, 1302, 1125, 780 cm^-1^; ^1^H-NMR (CDCl_3_) δ (ppm): 3.83 (s, 3H, CH_3_O), 7.00 (d, *J* = 8.2 Hz, 1H), 7.05 (d, *J* = 7.5 Hz, 1H), 7.22 (d, *J* = 8.2 Hz, 1H), 7.41 (m, 3H), 7.60 (m, 1H), 7.66 (s, 1H); ^13^C-NMR (CDCl_3_) δ (ppm): 55.7, 111.3, 116.7, 118.2, 120.6, 121.0, 126.9, 130.0, 130.4, 130.7, 131.0, 133.8, 140.2, 152.2, 156.9, 157.1; MS m/z (%): 288 ([M+2]^+^, 33), 286 (M^+^, 100), 269(16), 251 (8), 240 (5), 193 (21), 152 (16), 118 (14); Anal. Calcd for C_16_H_11_ClO_3_: C, 67.03; H, 3.87. Experimental: C, 67.25; H, 3.99.

*6-Chloro-3-(3’-methoxy)phenylcoumarin* (**7**). Purified by chromatography using 9:1 hexane/ethyl acetate as eluent; Mp: 144–146 °C; IR (KBr): 2925, 1700, 1615, 1300, 1115, 775 cm^-1^; ^1^H-NMR (CDCl_3_) δ (ppm): 3.85 (s, 3H, CH_3_O), 6.96 (dd, *J* = 2.5; 8.2 Hz, 1H), 7.25 (m, 2H), 7.35 (m, 2H), 7.48 (m, 2H), 7.72 (s, 1H); ^13^C-NMR (CDCl_3_) δ (ppm): 55.32, 114.2, 114.8, 117.8, 120.6, 120.8, 127.0, 129.2, 129.5, 129.7, 131.3, 135.5, 138.5, 151.8, 159.5, 159.8; MS m/z (%): 288 ([M+2]^+^, 29), 286 (M^+^, 100), 258(30), 215 (20), 201 (5), 152 (7); Anal. Calcd for C_16_H_11_ClO_3_: C, 67.03; H, 3.87. Experimental: C, 67.20; H, 3.95.

*6-Chloro-3-(4’-methoxy)phenylcoumarin* (**8**). Purified by chromatography using 9:1 hexane/ethyl acetate as eluent; Mp: 194–196 °C; IR (KBr): 2918, 1710, 1614, 1300, 1117, 779 cm^-1^; ^1^H-NMR (CDCl_3_) δ (ppm): 3.85 (s, 3H, CH_3_O), 6.99 (d, *J* = 8.9 Hz, 2H), 7.29 (d, *J* = 8.0 Hz, 1H), 7.48 (m, 2H), 7.66 (d, *J* = 8.9 Hz, 2H), 7.71 (s, 1H); ^13^C-NMR (CDCl_3_) δ (ppm): 55.4, 113.9 (2C), 117.7, 118.3, 120.3, 121.1, 126.8, 129.8 (2C), 130.8, 136.9, 141.1, 151.9, 156.3, 158.2; MS m/z (%): 288 ([M+2]^+^, 37), 286 (M^+^, 100), 258(13), 243 (16), 180 (3), 152 (5); Anal. Calcd for C_16_H_11_ClO_3_: C, 67.03; H, 3.87. Experimental: C, 67.10; H, 3.99.

*6-Bromo-3-(2’-methoxy)phenylcoumarin* (**9**). Purified by chromatography using 9:1 hexane/ethyl acetate as eluent; Mp: 183–185 °C; IR (KBr): 2909, 1709, 1615, 1305, 1120, 783 cm^-1^; ^1^H-NMR (CDCl_3_) δ (ppm): 3.82 (s, 3H, CH_3_O), 6.99 (d, *J* = 7.5 Hz, 1H), 7.03 (d, *J* = 7.4 Hz, 1H), 7.24 (d, *J* = 8.4 Hz, 1H), 7.36 (m, 2H), 7.60 (m, 2H), 7.66 (s, 1H); ^13^C-NMR (CDCl_3_) δ (ppm): 55.7, 111.3, 116.7, 118.2, 120.6, 121.0, 123.5, 127.6, 130.0, 130.5, 130.7, 133.8, 140.2, 152.5, 157.1, 159.6; MS m/z (%): 332 ([M+2]^+^, 100), 330 (M^+^, 97), 315(23), 237 (32), 152 (15), 118 (16); Anal. Calcd for C_16_H_11_BrO_3_: C, 58.03; H, 3.35. Experimental: C, 58.25; H, 3.49.

*6-Bromo-3-(3’-methoxy)phenylcoumarin* (**10**). Purified by chromatography using 9:1 hexane/ethyl acetate as eluent; Mp: 148-150 °C; IR (KBr): 2898, 1700, 1620, 1300, 1110, 789 cm^-1^; ^1^H-NMR (CDCl_3_) δ (ppm): 3.85 (s, 3H, CH_3_O), 6.96 (dd, *J* = 2.3; 8.2 Hz, 1H), 7.20 (m, 2H), 7.36 (m, 2H), 7.60 (d, *J* = 8.3 Hz, 1H), 7.75 (s, 1H), 7.85 (s, 1H); ^13^C-NMR (CDCl_3_) δ (ppm): 55.2, 114.1, 114.6, 116.9, 118.0, 120.7, 121.0, 129.0, 129.4, 130.0, 133.9, 135.3, 138.3, 152.1, 159.3, 159.6; MS m/z (%): 332 ([M+2]^+^, 100), 330 (M^+^, 28), 302(34), 195 (10), 180 (10), 152 (17); Anal. Calcd for C_16_H_11_BrO_3_: C, 58.03; H, 3.35. Experimental: C, 58.15; H, 3.50.

*6-Bromo-3-(4’-methoxy)phenylcoumarin* (**11**). Purified by chromatography using 9:1 hexane/ethyl acetate as eluent; Mp: 205–206 °C; IR (KBr): 2900, 1712, 1612, 1298, 1110, 781 cm^-1^; ^1^H-NMR (CDCl_3_) δ (ppm): 3.85 (s, 3H, CH_3_O), 6.97 (d, *J* = 8.8 Hz, 2H), 7.23 (m, 1H), 7.57 (dd, *J* = 2.2, 8.7 Hz, 1H), 7.63 (m, 3H), 7.67 (s, 1H); ^13^C-NMR (CDCl_3_) δ (ppm): 55.3, 113.9 (2C), 114.1, 116.9, 118.1, 121.4, 126.5, 128.9, 129.8 (2C), 129.9, 133.6, 136.8, 152.0, 160.4; MS m/z (%): 332 ([M+2]^+^, 100), 330 (M^+^, 13), 302(12), 288 (40), 259 (18), 195 (10), 180 (17), 152 (14); Anal. Calcd for C_16_H_11_BrO_3_: C, 58.03; H, 3.35. Experimental: C, 58.15; H, 3.42.

*6-Chloro-3-(2’-hydroxy)phenylcoumarin* (**12**). Purified by chromatography using 8:2 hexane/ethyl acetate as eluent. Mp: 225–227 °C. IR (KBr): 3500, 2920, 1705, 1613, 1300, 1120, 780 cm^-1^. ^1^H NMR (DMSO-*d_6_*) δ (ppm): 6.86 (m, 2H), 7.23 (m, 2H), 7.39 (d, *J* = 8.8 Hz, 1H), 7.78 (dd, *J* = 2.3; 8.8 Hz, 1H), 7.97 (s, 1H), 8.00 (s, 1H), 9.64 (bs, 1H, 1OH). ^13^C NMR (DMSO-*d_6_*) δ (ppm): 115.7, 116.0, 118.2, 118.7, 121.2, 121.8, 127.1, 129.9, 130.3, 130.7, 133.8, 140.6, 140.7, 152.1, 155.1. MS m/z (%): 274 ([M+2]^+^, 23), 272 (M^+^, 100), 244(35), 180 (19), 152 (20), 118 (10). Anal. Calcd for C_15_H_9_ClO_3_: C, 66.07; H, 3.33. Experimental: C, 66.25; H, 3.49.

*6-Chloro-3-(3’-hydroxy)phenylcoumarin* (**13**). Purified by chromatography using 8:2 hexane/ethyl acetate as eluent; Mp: 221–223 °C; IR (KBr): 3502, 2925, 1701, 1614, 1310, 1110, 785 cm^-1^; ^1^H-NMR (DMSO-*d_6_*) δ (ppm): 6.82 (dd, *J* = 2.2; 8.0 Hz, 1H), 7.10 (m, 2H), 7.25 (m, 1H), 7.44 (d, *J* = 8.8 Hz, 1H), 7.61 (dd, *J* = 2.5; 8.8 Hz, 1H), 7.86 (d, *J* = 2.5 Hz, 1H), 8.14 (s, 1H), 9.60 (bs, 1H, 1OH); ^13^C- NMR (DMSO-*d_6_*) δ (ppm): 115.5, 115.9, 117.8, 119.2, 120.9, 127.6, 128.0, 128.2, 129.3, 131.1, 135.5, 139.1, 151.5, 157.1, 159.2; MS m/z (%): 274 ([M+2]^+^, 21), 272 (M^+^, 100), 244(48), 180 (14), 152 (23), 118 (10); Anal. Calcd for C_15_H_9_ClO_3_: C, 66.07; H, 3.33. Experimental: C, 66.30; H, 3.45.

*6-Chloro-3-(4’-hydroxy)phenylcoumarin* (1**4**). Purified by chromatography using 8:2 hexane/ethyl acetate as eluent; Mp: 241–243 °C; IR (KBr): 3500, 2918, 1708, 1610, 1296, 1119, 783 cm^-1^; ^1^H-NMR (DMSO-*d_6_*) δ (ppm): 6.84 (d, *J* = 8.6 Hz, 2H), 7.44 (d, *J* = 8.8 Hz, 1H), 7.58 (m, 3H), 7.84 (d, *J* = 2.5 Hz, 1H), 8.09 (s, 1H), 9.81 (bs, 1H, 1OH); ^13^C-NMR (DMSO-*d_6_*) δ (ppm): 115.1 (2C), 117.7, 121.1, 124.8, 127.2, 127.8, 128.1, 129.9 (2C), 130.6, 137.1, 151.2, 158.2, 159.5; MS m/z (%): 274 ([M+2]^+^, 20), 272 (M^+^, 100), 244(74), 180 (15), 152 (40), 118 (5); Anal. Calcd for C_15_H_9_ClO_3_: C, 66.07; H, 3.33. Experimental: C, 66.22; H, 3.47.

*6-Bromo-3-(2’-hydroxy)phenylcoumarin* (**15**). Purified by chromatography using 8:2 hexane/ethyl acetate as eluent; Mp: 230–232 °C; IR (KBr): 3510, 2918, 1709, 1620, 1310, 1120, 780 cm^-1^; ^1^H-NMR (DMSO-*d_6_*) δ (ppm): 6.87 (m, 2H), 7.23 (m, 2H), 7.40 (d, *J* = 8.8 Hz, 1H), 7.74 (dd, *J* = 2.3; 8.8 Hz, 1H), 7.99 (s, 1H), 8.02 (s, 1H), 9.64 (bs, 1H, 1OH); ^13^C-NMR (DMSO-*d_6_*) δ (ppm): 115.7, 116.0, 118.2, 118.7, 121.2, 121.8, 127.1, 129.9, 130.3, 130.7, 133.8, 140.6, 152.1, 155.1, 158.8; MS m/z (%): 318 ([M+2]^+^, 47), 316 (M^+^, 71), 300(8), 288 (40), 180 (54), 152 (100); Anal. Calcd for C_15_H_9_BrO_3_: C, 56.81; H, 2.86. Experimental: C, 56.95; H, 3.00.

*6-Bromo-3-(3’-hydroxy)phenylcoumarin* (**16**). Purified by chromatography using 8:2 hexane/ethyl acetate as eluent; Mp: 219–221 °C; IR (KBr): 3505, 2919, 1709, 1622, 1312, 1123, 780 cm^-1^; ^1^H-NMR (DMSO-*d_6_*) δ (ppm): 6.82 (dd, *J* = 2.0; 7.7 Hz, 1H), 7.10 (m, 2H), 7.25 (m, 1H), 7.38 (d, *J* = 8.8 Hz, 1H), 7.73 (dd, *J* = 2.3; 8.8 Hz, 1H), 8.00 (d, *J* = 2.3 Hz, 1H), 8.14 (s, 1H), 9.59 (bs, 1H, 1OH); ^13^C- NMR (DMSO-*d_6_*) δ (ppm): 115.5, 115.9, 116.1, 118.1, 119.2, 121.4, 128.0, 129.3, 130.6, 133.9, 135.5, 139.0, 151.9, 157.1, 159.1; MS m/z (%): 318 ([M+2]^+^, 46), 316 (M^+^, 79), 288 (46), 180 (19), 152 (100); Anal. Calcd for C_15_H_9_BrO_3_: C, 56.81; H, 2.86. Experimental: C, 56.98; H, 3.01.

*6-Bromo-3-(4’-hydroxy)phenylcoumarin* (**17**). Purified by chromatography using 8:2 hexane/ethyl acetate as eluent; Mp: 226–228 °C; IR (KBr): 3520, 2939, 1710, 1622, 1302, 1133, 784 cm^-1^; ^1^H-NMR (DMSO-*d_6_*) δ (ppm): 6.84 (d, *J* = 8.8 Hz, 2H), 7.34 (d, *J* = 9.1 Hz, 1H), 7.54 (d, *J* = 8.8 Hz, 2H), 7.66 (m, 1H), 7.99 (s, 1H), 8.09 (s, 1H), 9.81 (bs, 1H, 1OH); ^13^C-NMR (DMSO-*d_6_*) δ (ppm): 115.1 (2C), 116.0, 118.0, 121.6, 124.8, 127.7, 129.9 (2C), 130.2, 133.3, 137.0, 151.6, 158.2, 159.4; MS m/z (%): 318 ([M+2]^+^, 31), 317 ([M+1]^+^, 100), 316 (M^+^, 40), 288 (39), 180 (14), 152 (46); Anal. Calcd for C_15_H_9_BrO_3_: C, 56.81; H, 2.86. Experimental: C, 56.87; H, 3.0.

### 3.3. Vasorelaxant activity

Vascular rings were prepared from aortae of male or female Wistar rats weighing 230–270 g. After an equilibration period of at least 1 h, isometric contractions induced by PE (1 μM) were obtained. When contraction of the tissue in response to the corresponding vasoconstrictor agent had stabilized (after about 20 min), cumulatively increasing concentrations of the compounds were added to the bath at 15–20 min intervals (the time needed to obtain steady-state relaxation). Control tissues were subjected to the same procedure simultaneously, but in this case omitting the compounds and adding the vehicle [appropriate dilutions of dimethylsulfoxide (DMSO)]. Results shown in the tables are expressed as means ± SEM from five experiments. Means were compared by one-way analysis of variance (ANOVA) followed by Dunnett’s post hoc test. The inhibitory effects of the tested compounds in rat aorta and human platelet are expressed as IC50 (concentrations that produce a 50% inhibition) estimated by least-squares linear regression using the program Origin 5.0, with *X* = log molar concentration of the tested compounds and *Y* = % of pharmacological response.

### 3.4. Antiplatelet activity

*Preparation of washed platelets*. Washed human platelets were prepared from blood anticoagulated with citrate-phosphate-dextrose, which was obtained from Centro de Transfusion de Galicia (Santiago de Compostela, Spain). Bags containing buffy coat from individual donors were diluted with the same volume of washing buffer (NaCl, 120 mM; KCl, 5 mM; trisodium citrate, 12 mM; glucose, 10 mM; sucrose, 12.5 mM; pH 6) and centrifuged at 400 g for 9 min. The upper layer containing platelet (platelet-rich plasma) was removed and centrifuged at 400 g for 9 min. The resulting platelet pellet was recovered, resuspended with washing buffer, and centrifuged again at 1,000 g for 15 min. Finally, the platelet pellet from this step was resuspended in a modified Tyrode-HEPES buffer (HEPES, 10 mM; NaCl, 140 mM; KCl, 3 mM; MgCl_2_, 0.5 mM; NaHCO_3_, 5 mM; glucose, 10 mM; pH 7.4) to afford a cell density of 2.5–3.5 × 10^8^ platelet/mL. The calcium concentration in the extracellular medium was 2 mM. 

*Platelet aggregation studies.* Platelet aggregation was measured using a dual channel aggregometer (Chrono-log, Havertown, PA, USA). Each tested compound, dissolved in DMSO, was incubated with washed platelet at 37 °C for 5 min. Stimulus (thrombin) was then added to induce platelet aggregation and the light transmission was monitored over 5 min period. Platelet aggregation is measured as the maximum change in light transmission during this period. The 100% aggregation value was obtained when vehicle (DMSO) was added instead of the compounds. The final DMSO concentration was below 1% (v/v) in all cases. 

## 4. Conclusions

In conclusion, some of the new synthesized molecules have been characterized as agents with remarkable human platelet antiaggregatory activity and significant vasorelaxant effects in intact rat aorta. Further experiments are in progress aimed at providing new data to clarify the precise mechanism by which coumarin-resveratrol hybrid derivatives produce their characteristic vasorelaxant and platelet antiaggregatory effects.

## Figures and Tables

*Reagents and conditions*: (a) DCC, DMSO, 110 °C, 12h; (b) HI/AcOH/Ac_2_O, 0 °C to rt, 3 h.

**Table 1:** Vasorelaxant activity (IC_50_ in μM) of tested compounds.

Compound	PE (1 μM)
**12**	36.63 ± 2.46^*^
**13**	57.63 ± 3.87^*^
**14**	**
**15**	48.79 ± 3.27^*^
**16**	46.67 ± 3.13^*^
**17**	**
***t*-RESV**	3.12 ± 0.26

^*^ P < 0.01 versus the corresponding IC_50_ values of *t*-RESV; ** Inactive at 100 μM (highest concentration tested). At higher concentrations compounds precipitate.

**Table 2:** Antiplatelet activity (IC_50_ in μM) for tested compounds.

Compound	Thrombin (0.25 U/mL)
**12**	91.36 ± 6.13^*^
**13**	6.41 ± 2.15^*^
**14**	**
**15**	**
**16**	**
**17**	20.1 ± 1.35^*^
***t*-RESV**	195.50 ± 13.82

^*^ P < 0.01 versus the corresponding IC_50_ values of *t*-RESV; ** Inactive at 100 μM (highest concentration tested). At higher concentrations compounds precipitate.
